# Association between non-high-density lipoprotein cholesterol to high-density lipoprotein cholesterol ratio and mortality in US adults: results from the NHANES 2011–2018

**DOI:** 10.3389/fnut.2025.1576229

**Published:** 2025-06-19

**Authors:** Mengmeng Yang, Junwei Zhang, Zhenyu Feng, Meiling Wang, Quanguan Feng, Runping Zhang, Chuanling Wu, Tian Wu, Genggeng Zhang, Yuanzhi Niu, Qiuju Dong, Qinghua Han, Tao Lin

**Affiliations:** ^1^The Third Clinical College, Shanxi University of Chinese Medicine, Taiyuan, China; ^2^School of Management, Shanxi Medical University, Jinzhong, China; ^3^Department of Hospital President, Shanxi Provincial Integrated TCM and WM Hospital, Taiyuan, China; ^4^Department of Scientific Research and Education, Shanxi Provincial Integrated TCM and WM Hospital, Taiyuan, China; ^5^Preventive Treatment Department of Traditional Chinese Medicine, Shanxi Provincial Integrated TCM and WM Hospital, Taiyuan, China; ^6^Department of Geriatrics, Shanxi Provincial Integrated TCM and WM Hospital, Taiyuan, China; ^7^Department of Medical Administration, Shanxi Provincial Integrated TCM and WM Hospital, Taiyuan, China; ^8^Department of Hospital President, The First Hospital of Shanxi Medical University, Taiyuan, China

**Keywords:** NHHR, mortality, NHANES, cross-sectional study, cardiovascular disease

## Abstract

**Background:**

The NHHR, which is the ratio of non-high-density lipoprotein cholesterol (non-HDL-C) to high-density lipoprotein cholesterol (HDL-C), has been suggested to have a link to several metabolic diseases and cardiovascular diseases (CVD). However, its association with CVD mortality and all-cause mortality remains uncertain.

**Methods and results:**

Analyzing HDL-C and non-HDL-C used to calculate NHHR were sourced from the National Health and Nutrition Examination Survey (2011–2018). In this experiment, we utilized subgroup analysis to examine the robustness of the results obtained. This study excluded participants under 20 years of age and those with missing NHHR or mortality data, resulting in a final sample of 20,294 participants. We employed logistic regression models to assess the association between NHHR and all-cause mortality and CVD mortality. The NHHR was categorized into four groups (Q1–Q4) according to their values from small to large. In model 3, considering all covariates, individuals in Q4 of NHHR exhibited a 41% higher CVD mortality compared to Q1 (HR = 1.41, 95% CI 1.14, 1.74). To explore the potential non-linear relationships between NHHR and mortality, restricted cubic spline (RCS) and threshold saturation techniques were used. About NHHR and all-cause mortality, the results indicated an L-shaped association, with a breakpoint (K) at 1.18. To the left of this threshold, a negative association was identified (OR = 0.30, 95% CI 0.13, 0.67). But when NHHR is greater than 1.18, the relationship was positive (HR = 1.12, 95% CI 1.06, 1.18).

**Conclusion:**

All in all, maintaining NHHR within an optimal range in adults may help reduce the association connected with both all-cause and CVD mortality.

## 1 Background

Approximately 48.6% of adults have cardiovascular disease (CVD), which is gradually overtaking cancer as one of the leading risks for mortality (1). The major cause of CVD is coronary artery disease, and prevention of coronary artery disease always takes into account the therapeutic prevention of dyslipidemia. Lipoprotein cholesterol (LDL-C) was always regarded as a major lipid target (2, 3). However, some clinical studies have shown that high triglycerides (TGs) and reduced high-density lipoprotein cholesterol (HDL-C) both promote the development of coronary heart disease (CHD) when LDL is controlled to low levels (4). Non-HDL-C is the cholesterol in many types of proatherogenic lipoproteins that carry apolipoprotein B100 (ApoB100) (5). Therefore, a number of reports have noted that it was proposed that non-HDL was a common target with LDL for lipid management or that non-HDL-C may be considered a primary target (6–8).

In comparison to traditional lipid indicators, non-high-density lipoprotein cholesterol to high-density lipoprotein cholesterol ratio (NHHR) provides a more thorough and persuasive indicator (9). Recently, the NHHR has shown great advantage in evaluating diseases, including thyroid hormones (10), uric acid (11), diabetes (12, 13), and cardiovascular disease (14, 15). Because there is limited research examining the relationship between NHHR and mortality, this area remains underexplored. To address this issue, explicit the association between NHHR and mortality by using the data of 2011–2018 National Health and Nutrition Examination Survey (NHANES).

## 2 Methods

### 2.1 NHANES

The NHANES is a continuous, cross-sectional study conducted by the National Center for Health Statistics (NCHS). It employs a complex, multistage probability sampling design to obtain a nationally representative sample of the non-institutionalized civilian population in the United States. The survey includes health interviews, physical examinations, and laboratory tests.

### 2.2 Criteria for participant enrollment

**Figure 1:**
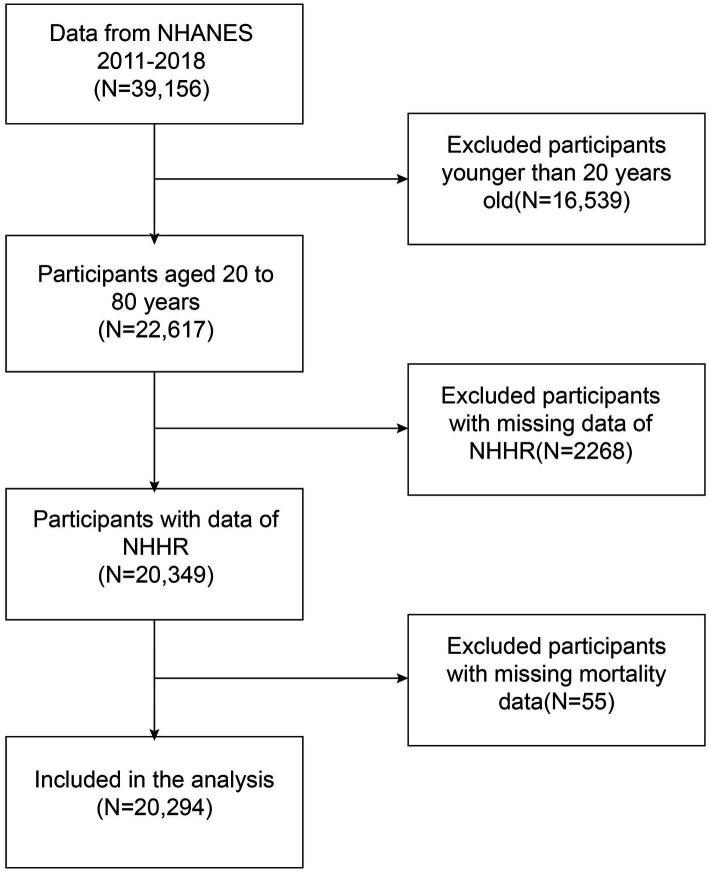
Flowchart depicting participant selection from the National Health and Nutrition Examination Survey (NHANES) (2011–2018).

The study enrolled 20,294 participants, who were recruited during the 2011–2018 NHANES cycle. Exclusion criteria are as follows: the age of participants less than 20 (*n* = 16,539); missing information on NHHR data (*n* = 2,268); missing information on mortality information (*n* = 55). These screening steps are illustrated in the Figure 1.

### 2.3 Exposure factor

The NHHR was derived from TC and HDL-C lipid screenings done by the participants. Non-HDL-C represents the cholesterol content of all proatherogenic lipoproteins, which include: low-density lipoprotein cholesterol (LDL-C); very low-density lipoprotein cholesterol (VLDL-C); intermediate-density lipoprotein cholesterol (IDL-C); lipoprotein (a) [Lp(a)], and chylomicron remnants. Non-HDL-C is calculated by subtracting high-density lipoprotein cholesterol (HDL-C) from total cholesterol (TC). Non-HDL-C = TC − HDL-C. Finally, NHHR was calculated by dividing non-HDL-C by HDL-C (16).

### 2.4 Primary outcome

The event endings of the study were all-cause and cardiovascular deaths. All-cause deaths were obtained from NCHS. The files integrate survey data with mortality information from the National Death Index (NDI), a comprehensive database. The resource can analyze the trends in mortality across different demographic groups, providing valuable insights into causes of death and public health concerns. The cause of death was determined by the International Classification of Diseases 10th revision (ICD-10) coding. All-cause mortality encompassed deaths from any cause. Cardiovascular mortality refers to diseases of the heart (I00-I09, I11, I13, and I20-I51).

### 2.5 Covariates

Covariates, including demographic, anthropometric, and laboratory-based variables, were collected through standardized questionnaires, physical examinations, questionnaires, and laboratory tests. Covariates based on previous research, a series of covariates that may be confounding, were summarized in adjusted models (12, 17, 18). The covariables have the following components of indicators. Demographic covariates included sex, age, race, education level, poverty income ratio (PIR), and marital status.

In addition, anthropometric measurements and laboratory-based variables were also included as covariates, such as body mass index (BMI, kg/m^2^), total cholesterol (TC, mg/dL), GHB (glycated hemoglobin, %), and FBG (fasting blood glucose, mmol/L). The health condition is influenced by smoking status, which is classified into two categories: smokers and non-smokers. Smoking status is determined based on whether an individual has smoked more than 100 cigarettes in their lifetime.

### 2.6 Statistical analysis

First, we divided NHHR into four groups and regarded the lowest quartile (Q1) as the reference category. We employed logistic regression models to assess the association between NHHR and mortality outcomes and constructed three models adjusting for various covariates. Model 1 was unadjusted, meaning no covariates were included. Model 2 was adjusted partially to account for age, sex, and race. Based on Model 2, Model 3 incorporated education level, smoking status, marital status, PIR, BMI, TC, FBG, and GHB. In this experiment, subgroup analyses were performed to explore potential interactions. To further investigate whether there was a non-linear relationship between NHHR and either CVD or all-cause mortality, restricted cubic spline (RCS) and threshold saturation techniques were applied. In addition, subgroup analyses were performed, stratified by age, sex, BMI, and smoking status, to assess the robustness of the results. All statistical analyses were conducted using EmpowerStats version 2.0 and R version 4.3.1.

## 3 Results

### 3.1 Baseline characteristics

Of our participants, including 20,294 participants, about 48.3% were male. In total, the overall prevalence of all-cause mortality was 6.2%, and of the NHHR quartile grouping, group 3 had the highest all-cause mortality rate. According to Table 1, the average NHHR was 2.89 ± 1.14. The NHHR values for the four quartiles (Q1 to Q4) ranged as follows: Q1 (1.87–2.21), Q2 (1.88–2.60), Q3 (2.61–3.53), and Q4 (3.54–26.85). In the Table 1, participants in the highest NHHR quartile (Q4) were more likely to be older (40–60 years), male, obese (BMI ≥ 30 kg/m^2^), in education college or above, married/living with partner, and have higher fasting glucose, TC, and glycated hemoglobin levels compared to those in the lowest quartile (Q1). These differences were statistically significant (*p* < 0.05).

**Table 1:** Baseline characteristics according to NHHR quartiles.

Characteristics	Q1 (2.21–1.87)	Q2 (1.88–2.6)	Q3 (2.61–3.53)	Q4 (3.54–26.85)	*p*-value
	*N* = 5,042	*N* = 5,080	*N* = 5,084	*N* = 5,088	
Age (years), *n* (%)					0.002
20–40	1900(37.68)	1727(34.00)	1,548(30.45)	1,571(30.88)	
40–60	1,302(25.82)	1,513(29.78)	1831(36.01)	2074(40.76)	
60–80	1840(36.49)	1840 (36.22)	1705(33.54)	1,443(28.36)	
Sex, *n* (%)					<0.001
Male	1790(35.50)	2,150(42.32)	2,586(50.87)	3,285(64.56)	
Female	3,252(64.50)	2,930(57.68)	2,498(49.13)	1803(35.44)	
Race, *n* (%)					<0.001
Mexican American	507(10.06)	636(12.52%)	768(15.11)	892(17.53)	
Non-Hispanic White	1894(37.56)	1881(37.03)	1911(37.59)	1921(37.76)	
Non-Hispanic Black	1,432(28.40)	1,218(23.98)	1,031(20.28)	778(15.29)	
Other	1,209(23.98)	1,345(26.48)	1,374(27.03)	1,497(29.42)	
Education, *n* (%)					<0.001
Less than high school	925(18.35)	1,037(20.41)	1,156(22.74)	1,340(26.34)	
High school grad or equivalent	1,061(21.04)	1,103(21.71)	1,183(23.27)	1,159(22.78)	
Some college or above	3,050(60.49)	2,938(57.83)	2,740(53.89)	2,583(50.77)	
other	6(0.12)	2(0.04)	5(0.10)	6(0.12)	
Marital, *n* (%)					
Married/living with partner	2,660(52.76)	2,915(57.38)	3,093(60.84)	3,304(64.94)	<0.001
Widowed/divorced/separated	1,174(23.28)	1,150(22.64)	1,121(22.05)	1,017(19.99)	
Never married	1,208(23.96)	1,015(19.98)	864(16.99)	763(15.00)	
Living with partner	0(0.00)	0(0.00)	6(0.12)	4(0.08)	
PIR, *n* (%)					<0.001
Low	1,374(27.25)	1,415(27.85)	1,527(30.04)	1,652(32.47)	
Moderate	2,150(42.64)	2,224(43.78)	2,239(44.04)	2,192(43.08)	
High	1,518(30.11)	1,441(28.37)	1,318(25.92)	1,244(24.45)	
BMI (kg/m^2^), *n* (%)					<0.001
Normal	2,391(47.42)	1,587(31.24)	1,063(20.91)	664(13.05)	
Overweight	1,474(29.23)	1,665(32.78)	1764(34.70)	1819(35.75)	
Obese	1,177(23.34)	1828(35.98)	2,257(44.39)	2,605(51.20)	
Smoking status, *n* (%)					<0.001
Yes	2003(39.73)	2014(39.65)	2,151(42.31)	2,498(49.10)	
No	3,034(60.17)	3,062(60.28)	2,929(57.61)	2,588(50.86)	
TC (mmol/L), mean (SD)	4.33 ± 0.90	4.67 ± 0.90	4.98 ± 0.90	5.68 ± 1.11	<0.001
GHB (%)	5.60 ± 0.88	5.72 ± 0.96	5.85 ± 1.10	6.06 ± 1.41	<0.001
FBG (mmol/L)	5.70 ± 1.15	5.80 ± 1.27	5.90 ± 1.39	6.09 ± 1.90	<0.001

Mean ± SD for continuous variables: the *p*-value was calculated by the linear regression model; for categorical variables: the *p*-value was calculated by the chi-square test. SE, standard error; PIR, the ratio of income to poverty; BMI, body mass index (calculated as weight in kilograms divided by height in meters squared. BMI was categorized as follows: less than 25 was classified as normal weight, 25–29.9 kg/m^2^ as overweight, and 30 kg/m^2^ or higher as obese); Q, quartile; BMI, body mass index; TC, total cholesterol; FBG, fasting blood sugar; GHB, glycated hemoglobin.

### 3.2 Associations of NHHR and all-cause mortality

**Figure 2:**
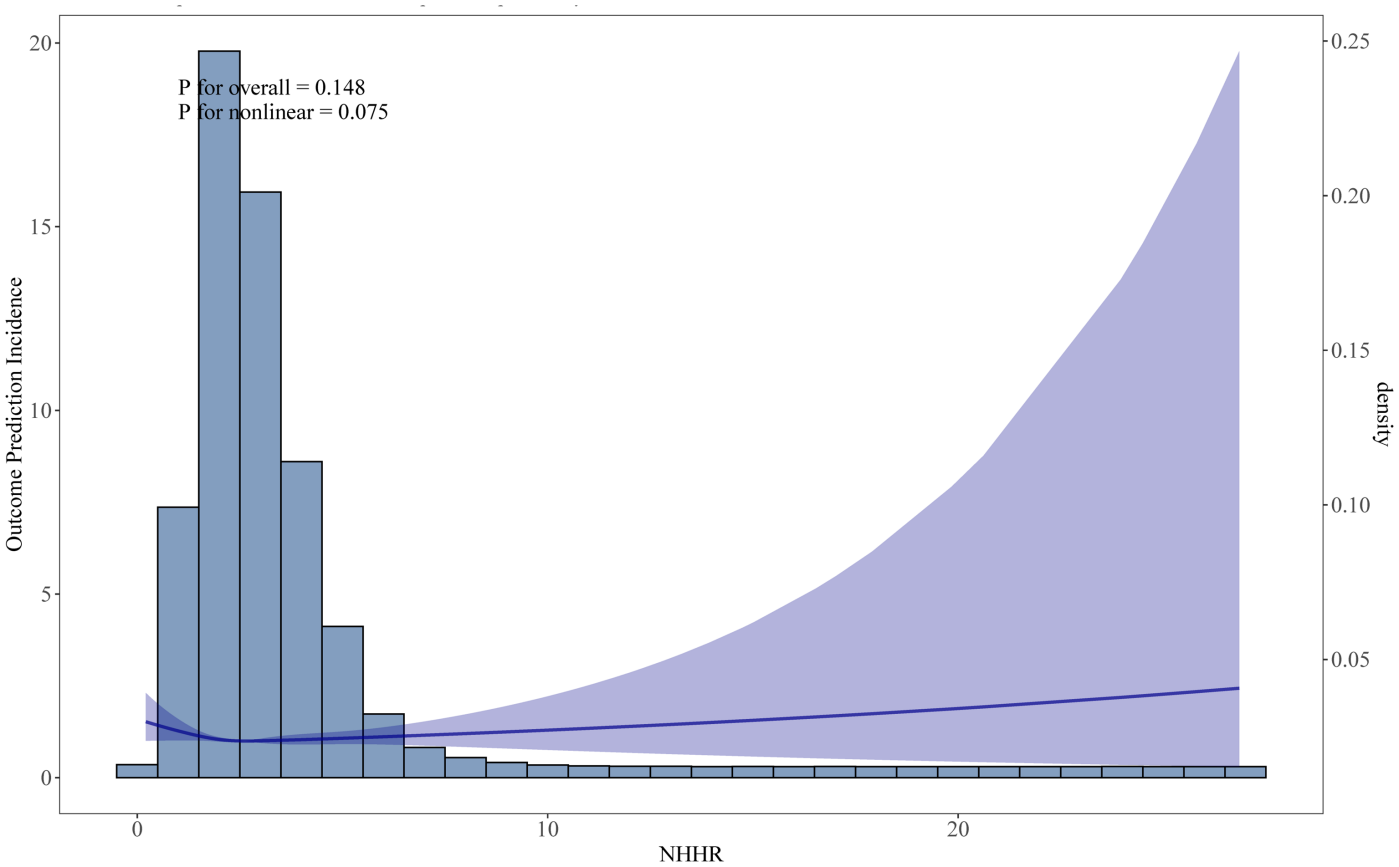
Restricted cubic spline (RCS) curve fit between NHHR and all-cause mortality. Solid lines represent smooth curve fits between variables. Shaded bands represent 95 percent confidence intervals from the fit.

In Table 2, the lower risk of Q4 when model 1 was not adjusted for covariates may have been caused by confounding factors such as a higher proportion of younger age groups. After model 3 adjusted for variables such as age and smoking, the independent effect of NHHR emerged, showing a significantly higher risk of Q4 (HR = 1.41), suggesting that covariates such as age, and metabolic markers had a significant impact on the results. In Figure 2, we identified a significant L-shaped relationship and in Table 3 determined the breakpoint (K) to be 1.18. To the left of this breakpoint, a negative relationship (HR = 0.30, 95% CI 0.13, 0.67) was detected. On the right, there was a positive relationship (HR = 1.12, 95% CI 1.06, 1.18).

**Table 2:** HRs (95% CIs) for mortality according to NHHR quartiles.

Cause of death	Quartiles of NHHR				*P* for trend
	Q1(0.21–1.87)	Q2(1.88–2.6)	Q3(2.61–3.53)	Q4(3.54–26.85)	
All-cause mortality					
Model 1 HR(95%CI) *p*-value	1.0	0.99 (0.84, 1.15) 0.86	0.81 (0.69, 0.95) 0.01	0.83 (0.71, 0.98) 0.03	0.008
Model 2 HR(95%CI) *P*-value	1.0	1.05 (0.89,1.24) 0.59	0.94 (0.79, 1.12) 0.51	1.16 (0.97, 1.38) 0.11	0.174
Model 3 HR(95%CI) *P-*value	1.0	1.14 (0.96,1.36) 0.13	1.03 (0.86, 1.25) 0.74	1.41 (1.14, 1.74) 0.01	0.005
CVD mortality					
Model 1 HR(95%CI) *p*-value	1.0	1.12 (0.80,1.59) 0.51	1.13 (0.79, 1.62) 0.51	1.33 (0.93, 1.89) 0.11	0.122
Model 2 HR(95%CI) *p*-value	1.0	1.14 (0.81,1.62) 0.45	1.21 (0.84, 1.75) 0.31	1.53 (1.07, 2.20) 0.02	0.020
Model 3 HR(95%CI) *p*-value	1.0	1.06 (0.74,1.52) 0.74	1.10 (0.75,1.64) 0.61	1.49 (1.97, 2.25) 0.06	0.005

Model 1: no covariates were adjusted.

Model 2: adjusted for sex, age, and race.

Model 3: adjusted for sex, age, race, education level, marital status, PIR, BMI, TC, FBG, Smoking status, and hemoglobin A1c.

β: effect size.

95% CI: 95% confidence interval.

HR: Hazard Ratio.

**Table 3:** Threshold effect analysis of NHHR on all-cause mortality in participants.

Threshold effect analysis	HR (95%CI), *p*-value
All-cause mortality	
Breakpoint (K)	1.18
NHHR (< 1.18)	0.30(0.13, 0.67) 0.0034
NHHR (> 1.18)	1.12(1.06, 1.18) 0.0001
Logarithmic likelihood ratio test *p*-value	0.002

The model was adjusted for sex, age, race, education level, Marital, PIR, body mass index, total cholesterol, fasting blood sugar and glycated haemoglobin.

CI, confidence interval; CVD, cardiovascular disease; HR, Hazard ratio.

### 3.3 Associations between NHHR and CVD mortality

**Figure 3:**
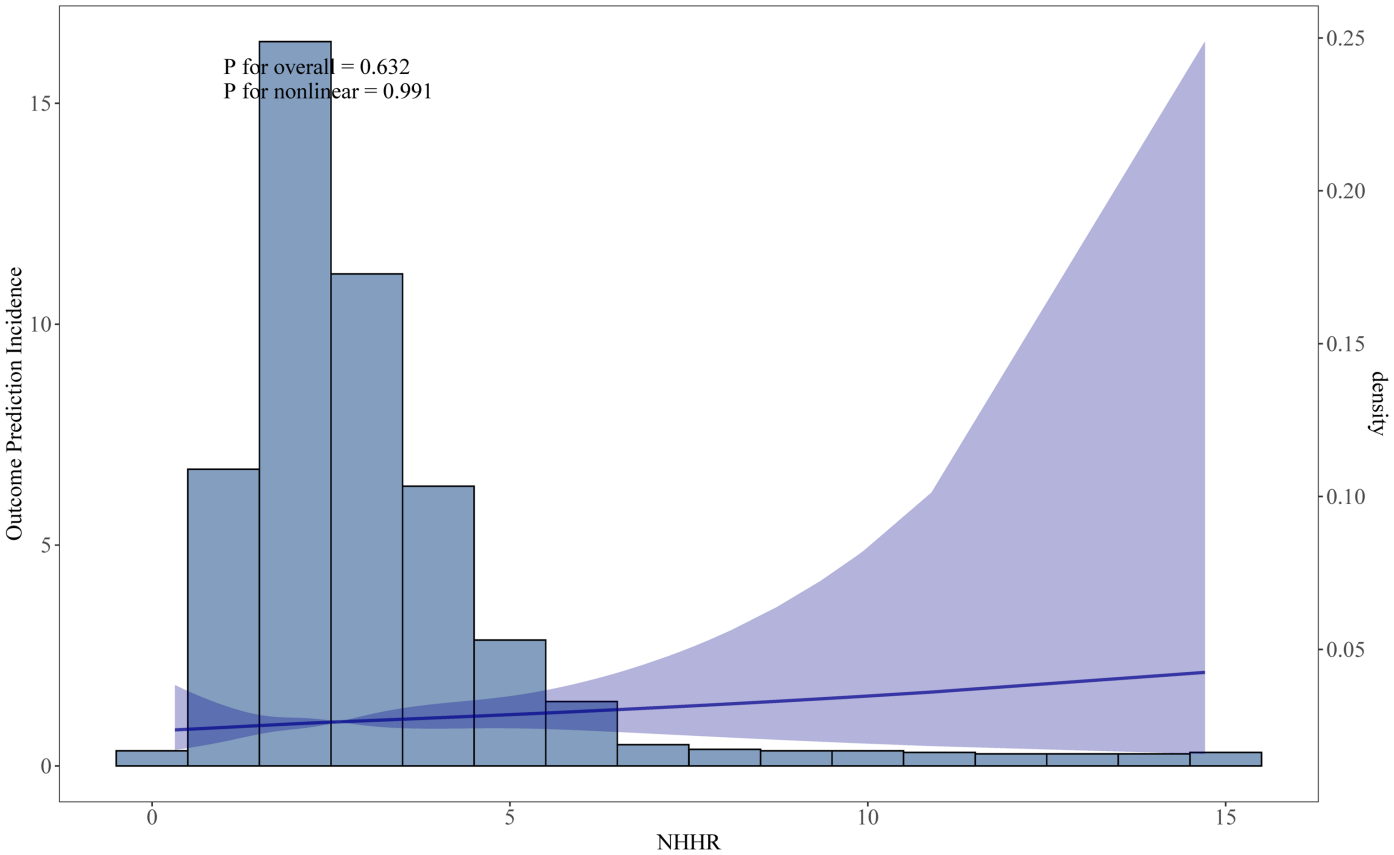
Restricted cubic spline curve fit between NHHR and CVD mortality. Solid lines represent smooth curve fits between variables. Shaded bands represent 95 percent confidence intervals from the fit.

During the survey period, a total of 1,259 participants passed away, with 335 of these deaths attributed to cardiovascular disease. In Table 2, the fully adjusted model (Model 3), participants in Q4 (HR = 1.49, 95% CI: 1.97–2.25) had a 49% higher CVD mortality compared to those in Q1. The RCS analysis in Figure 3 confirmed a linear relationship between NHHR and CVD mortality, suggesting that the likelihood increases steadily with higher NHHR values.

### 3.4 Subgroup analyses

To validate the potential association between NHHR and all-cause mortality, subgroup analyses were conducted to examine how variations in NHHR levels correlate with the death from any cause. The aim was to uncover potential disparities in the association between NHHR and within specific demographic contexts, view subgroup analysis in Table 4. These analyses, stratified by sex, age, BMI, and smoking status, revealed that the association remained consistent across all subgroups (*P* for interaction > 0.05).

**Table 4:** Subgroup analysis for the association between NHHR and all-cause mortality or CVD mortality.

Characteristics	CVD mortality [HR(95%CI)]	*P* for interaction	All-cause mortality [HR(95%CI)]	*P* for interaction
Age		0.346		0.719
20–40	1.18 (1.04, 1.35)		1.31 (0.91, 1.87)	
40–60	1.06 (0.94, 1.18)		1.14 (0.86, 1.52)	
≥60	1.05 (0.98, 1.13)		1.11 (0.97, 1.27)	
Gender		0.143		0.448
Male	1.04 (0.97, 1.12)		1.09 (0.95, 1.24)	
Female	1.13 (1.04, 1.24)		1.17 (1.00, 1.38)	
BMI (kg/m^2^)		0.587		0.816
Normal/underweight	1.13 (1.00, 1.27)		1.18 (0.91, 1.51)	
Overweight	1.02 (0.92, 1.12)		1.08 (0.89, 1.30)	
Obese	1.07 (0.98, 1.16)		1.07 (0.90, 1.27)	
Smoking status		0.301		0.883
Yes	1.05 (0.98, 1.13)		1.13 (0.98, 1.29)	
No	1.12 (1.02, 1.23)		1.11 (0.90, 1.36)	

HR, Hazard ratio.

## 4 Discussion

This article focused on the correlation between NHHR and mortality. Based on our cross-sectional analysis of 20,294 participants, about NHHR and all-cause mortality, the results indicated an L-shaped association. Additionally, the NHHR has an inflection point at 1.18. To the left of this breakpoint, a negative relationship (HR = 0.30, 95% CI 0.13, 0.67; *P* for trend = 0.0034) was detected. On the right, there was a positive relationship (HR = 1.12, 95% CI 1.06, 1.18; *P* for trend = 0.0001).

The NHHR is a composite lipid marker that reflects the balance between proatherogenic (non-HDL-C) and antiatherogenic (HDL-C) lipoproteins. Abnormalities in lipid metabolism, such as elevated non-HDL-C and reduced HDL-C, are known to contribute to the development of atherosclerosis and cardiovascular disease. You et al. (19) and Mao et al. (20) suggested that a positive association has been observed between lipid metabolism abnormalities and coronary artery disease. And that NHHR could be regarded as an independent predictor of adverse cardiovascular events. NHHR is superior to traditional lipid indices for predicting patients with ST-elevation myocardial infarction and may be possible (21). The results of another clinical study on slow coronary blood flow were similar (22). A study by Yu et al. (23), which included 12,578 participants, also found that NHHR and mortality had a non-linear relationship in diabetic patients. All these reports corroborate our study side by side.

Although there are few studies on NHHR and mortality, multiple studies have indicated a possible link of NHHR and the progression of atherosclerosis (16, 24, 25). The progression of atherosclerosis is linked to a range of underlying mechanisms, including endothelial dysfunction, inflammatory responses, lipid deposition, and changes in cellular phenotype. Among these mechanisms, the lipid hypothesis has gained increasing attention, with lipid deposition due to dyslipidemia playing a crucial role (18, 26). Elevated NHHR may represent abnormal lipid metabolism (27). There has been a strong correlation between non-HDL-C and cardiovascular events (28–31). With elevated non-HDL-C, cholesterol crystallization occurs in subendothelial immune cells, leading to lysosomal damage. This process subsequently triggers the activation of NLRP3 inflammasome complexes, leading to the enhanced release of IL-1β and IL-18, which further amplifies the inflammatory cascade (32). This inflammatory response exacerbates endothelial dysfunction and promotes foam cell formation, key steps in atherosclerotic plaque development (8). More notably, TLR activation (via LPS or oxidized LDL) inhibits LXR (Liver X Receptor) activity and reduces the expression of ABC subfamily A member 1 (ABCA1) and ABCG1 (33), thereby inhibiting cholesterol efflux (34). In addition, cholesterol accumulation leads to the formation of lipid rafts, and receptors such as TLR4 and TLR6 accumulate in lipid rafts on the cell membrane (35).

Activation of the TLR (Toll-like receptor) signaling pathway through the adaptor proteins MyD88 (Myeloid Differentiation Primary Response Protein 88) and TRIF (TIR-domain-containing adaptor-inducing interferon-β) results in the activation of nuclear factor-κB (NF-κB) and other transcriptional regulators, including interferon regulatory factor (IRF) (36, 37). This cascade drives the production of inflammatory mediators, such as TNF-*α*, IL-6, IL-1β, and various chemokines (38). This inhibition leads to further intracellular accumulation of cholesterol, creating a positive feedback loop that amplifies the inflammatory response.

In contrast to non-HDL-C, HDL-C promotes the expression of ABCA1 and ABCG1 (35), the key transporters responsible for the outflow of cholesterol from macrophages to HDL, by activating the liver X receptor (LXR) (34, 35, 39). When NHHR is elevated, reduced HDL-C levels inhibit LXR activity, leading to decreased expression of ABCA1 and ABCG1, leading to intracellular cholesterol accumulation and an enhanced inflammatory response (40). HDL is not only involved in reverse cholesterol transport, but also has antioxidant activity. HDL prevents the accumulation of lipid peroxides during LDL oxidation by inhibiting the activity of enzymes such as 12-lipoxygenase (41). Together, these molecular pathways illustrate how elevated NHHR contributes to the pathogenesis of atherosclerosis through inflammation and oxidative stress.

This study obtained a large sample size with a sample of 20,294 participants. The effect of confounders was included in this study based on relevant literature to ensure the reliability of the results of this experiment. Finally, analyzing the effects of confounding factors on NHHR and mortality by subgroup analysis. However, due to its cross-sectional design, the correlation and reliability of the observations are uncertain, and further assessment of their prediction of mortality through prospective cohort or mechanistic studies is needed to validate the clinical application of NHHR. Moreover, since the data was obtained from a publicly available dataset, the cross-sectional design could not be altered. Although we accounted for several covariates, other potential confounders might still have influenced the findings. For instance, certain variables that determine whether cardiovascular deaths are caused by a primary or secondary disease were not included in the NHANES dataset, restricting our ability to incorporate them into the study. Additionally, because NHANES was conducted in the United States, the results are only relevant to U.S. adults. Given the sample was limited to a single country and had restricted ethnic diversity, caution should be exercised when attempting to generalize these findings to other populations.

## 5 Conclusion

The findings propose novel ideas about the relationship between lipid metabolism abnormalities, CVD, and all-cause mortality, emphasizing the potential of the NHHR as an effective tool for clinical assessment. Healthcare providers can mitigate NHHR by identifying patients for CVD and implementing preventative strategies to enhance overall health.

## Data Availability

The datasets presented in this study can be found in online repositories. The names of the repository/repositories and accession number(s) can be found in the article/supplementary material.
